# High Performance Composites Based on Highly Filled Glass Fiber-Reinforced Polybenzoxazine for Post Application

**DOI:** 10.3390/polym14204321

**Published:** 2022-10-14

**Authors:** Phattarin Mora, Chananya Nunwong, Parkpoom Sriromreun, Preecha Kaewsriprom, Ukrit Srisorrachatr, Sarawut Rimdusit, Chanchira Jubsilp

**Affiliations:** 1 Department of Chemical Engineering, Faculty of Engineering, Srinakharinwirot University, Nakhonnayok 26120, Thailand; 2 Research Unit in Polymeric Materials for Medical Practice Devices, Department of Chemical Engineering, Faculty of Engineering, Chulalongkorn University, Bangkok 10330, Thailand; 3 Department of Mechanical Engineering, Faculty of Engineering, Srinakharinwirot University, Nakhonnayok 26120, Thailand; 4 Excellent Center for Sustainable Engineering, Faculty of Engineering, Srinakharinwirot University, Nakhonnayok 26120, Thailand; 5 Institute of Dentistry, Department of Medical Services, Nonthaburi 11000, Thailand

**Keywords:** polymer composites, resin composite, fracture resistance, fiber post, finite element analysis

## Abstract

Glass fiber post based on the new polymeric material, polybenzoxazine, is prepared and the effects of glass fiber contents on mechanical and thermal properties are evaluated. The mechanical response to externally applied loads of tooth restored with glass fiber-reinforced polybenzoxazine composite posts is also simulated by finite element analysis of a tridimensional model and compared with the response to that of a natural tooth. The reinforcing of glass fiber can help improve the mechanical and thermal properties of the polybenzoxazine influenced by the interfacial adhesion between the glass fiber and polybenzoxazine matrix, except for the relatively high mechanical property of the glass fiber. The mechanical data, i.e., elastic modulus under flexure load or flexural modulus by three-point bending test of the glass fiber-reinforced polybenzoxazine composites are agreed with the elastic modulus of dentin and then used in the finite element model. The restoration using the glass fiber-reinforced polybenzoxazine composite post provided the maximum von Mises equivalent stress at the cervical third area of the endodontically treated tooth model as similarly observed in the natural tooth. In addition, the maximum von Mises equivalent stress of the tooth restored with the glass fiber-reinforced polybenzoxazine composite post is also quietly like that of the natural tooth. The finding of this work provided the essential properties of the glass fiber-reinforced polybenzoxazine composite for dental restorations and appliances.

## 1. Introduction

Fiber-reinforced thermosetting polymer composites have been gaining tremendous potential for usage in numerous applications which demand excellent mechanical properties due to their light weight and superior specific strength and modulus, i.e., thermosetting polymer reinforced with carbon fiber (CFRP) and glass fiber (GFRP). CFRP can be used in some engineering with high bearing capacity and harsh corrosion environments due to its very excellent mechanical properties, fatigue resistance, and corrosion resistance even though it is a high price. While GFRP showed good mechanical properties with a low price, however, the long-term corrosion resistance and fatigue resistance are weak when facing some complex environmental exposure [1,2,3]. Over the past few years, the desire for fiber-reinforced polymer composites (non-metallic) has increased for dental fiber post applications such as carbon fiber, quartz fiber, and glass fiber posts to replace metallic posts due to the drawbacks of metallic posts, including their propensity for corrosion, root fractures, and loss of retention, as well as the rising desire for cosmetic restorations, all ceramic crowns must be supported by aesthetic posts [4,5,6,7,8,9,10,11]. In addition, desired properties of the post material, including mechanical properties, i.e., elastic modulus, compressive strength, flexural strength, and thermal expansion, should be close to that of dentin. Additionally, it should effectively bond to the dentin and resemble it cosmetically. There may be areas of concentrated stress in the dentin that could eventually cause root cracking and fracture, as in the case of zirconium and metal posts, because of the mismatch between the elastic modulus of the dentin and the selected post [12,13]. For bonded resin and ceramic restorations in the anterior region, the presence of a metal post can also result in the shadowing of the soft tissues next to the root surface [14]. In contrast, fiber-reinforced composite posts and fiber posts displayed a more esthetic material in this area. Furthermore, the fiber post has many benefits, including high fatigue resistance and a low elastic modulus that is comparable to dentin’s (18–40 GPa) [15,16], and excellent light conductivity. They distribute forces evenly in the root resulting in fewer root fractures when bonded with resin cement. Fiber posts can be easily removed with little to no tooth removal in the event that the endodontic treatment needs to be redone, allowing the dentist options for additional treatment [1,17].

The main component of fiber posts, which are composite materials, typically comprises a resin matrix/binder with a high cross-linked structure and a high degree of conversion [18,19]. The fibers could be manufactured of glass/silica, carbon, and quartz. Whereas the most popular resin bases are epoxy and bis-GMA (bisphenol A glycidyl methacrylate), and it has been suggested that polyimide might be used as a resin matrix for fiber posts [4,20]. In addition, polyetheretherketone (PEEK) and epoxy/polyimide used to produce fiber posts are presented [20,21]. However, among polymer matrices for the fiber posts, epoxy resins are notable and growing as several glass fiber posts consisting of glass fiber and epoxy resin are commercial [22,23,24]. The materials offer strength and toughness equivalent to dental tissues by experimental investigation, with very satisfactory aesthetics. The flexural properties and the micro-morphology of six types of glass/quartz fiber posts have been evaluated [25]. It was found that flexural modulus and flexural strength depended on the post’s diameter was in a range of 10–25 GPa, and 590–1090 MPa, respectively. When comparing the flexural strength of the post’s similar dimensions, the glass fiber/zirconia filler-filled epoxy resin post performed significantly better flexural strength than that of the other posts, i.e., glass fiber/dimethacrylates post, glass fiber/Bis-GMA post, and quartz fiber/epoxy resin post. In addition, the lower interfacial bonding between quartz fibers and epoxy resin matrix might result in lower flexural strength when compared with that of the glass fiber/zirconia filler-filled epoxy resin post. A comparison of the flexural properties of glass fiber posts and carbon fiber posts has also been studied [26]. The glass fiber posts showed their flexural modulus, i.e., 13–24 GPa being closer to that of the dentin when compared to the flexural modulus of carbon fiber posts, i.e., 8–51 GPa. While the flexural strength was quietly similar, i.e., 540–1130 MPa for glass fiber posts and 580–900 MPa for carbon fiber posts. In addition, the effect of physical parameters, including the amount of fiber (vol%), their dispersion index, and coordination number on flexural properties of five types of posts from three different manufacturers, i.e., quartz fiber/epoxy posts, zircon-rich glass fiber/epoxy post, zircon rich glass fiber/vinyl-polyester methacrylate post, and glass fiber/urethane methacrylate post have also been investigated [27]. The posts showed a flexural modulus of about 45.7–53.8 GPa and flexural strength in a range of 936.3–1889.6 MPa. They summarized that the weaker posts showed more fiber dispersion, higher resin contents, and larger numbers of visible defects. The flexural strength was inversely correlated with fiber diameter, while the flexural modulus was weakly related to coordination number, amount of fiber (vol%), and dispersion index. In addition, it was observed that the interfacial adhesion between the silica-based fibers and the resin matrix was to be of paramount importance. Moreover, as suggested by an amount of literature, numerical simulations using finite element analysis (FEA) were employed to evaluate more precise information about the stress distribution around endodontic posts, which also provided insight into the stress distribution inside the post. For example, different types of posts, i.e., glass fiber posts, carbon fiber posts, and cast metal posts used to restore teeth have been studied [28]. According to the investigators, due to their flexibility and use of less stiff core material, glass fiber and carbon fiber posts revealed very significant stresses in the cervical area, but the gold cast post and core created the greatest stress concentration at the post-dentin interface. The lowest stress inside the root for the tooth restored with glass fiber post was observed because its stiffness is much like dentin. In addition, the glass fiber post caused a stress field that was remarkably like that of a natural tooth, with the exception of the force concentration at the cervical margin. Another study using FEA compared the stress distribution in the dentin and cement layer of a maxillary incisor that had been repaired using glass fiber post, carbon fiber post, and steel post [29]. The results showed that the lowest of maximum von Mises equivalent stress belonged to a tooth stored with a glass fiber post, while the tooth stored with a steel post showed the highest maximum von Mises equivalent stress. This is due to steel posts and conventional cement, which lack adhesive and are more rigid than glass and carbon posts, as well as sticky resin cement, which does not provide a uniform distribution of stress.

However, the curing agents or hardeners employed for the epoxy resins, such as anhydrides and amines, have several disadvantages for glass fiber posts with epoxy-based matrices, such as poor storage stability or high toxicity. Epoxy resin also has the drawback of having poor heat resistance, as seen by its low glass transition temperature and modest thermal expansion [30,31], and they showed intrinsic brittleness due to its high crosslink density, which might cause its breakage during functioning [20]. In recent years, studies are also being conducted on alternative resins such as polyimide as potential replacements for the traditional components in the fiber post-resin base [32]. In addition, to overcome the utilization of highly toxic curing agent for epoxy resin and awareness of harmful industrial processes, polybenzoxazines, a novel type of phenolic, is a suitable alternative to epoxy resins as they show the excellent property balance of mechanical, thermal, electrical, chemical, and physical properties. They can also be prepared from inexpensive basic ingredients and polymerized by a ring-opening addition reaction with low a-stage viscosity and no reaction by-products. The extraordinary molecular design flexibility of polybenzoxazines enables the polymerized materials’ characteristics to be modified in a variety of ways to meet the demands of various applications [33,34,35,36,37].

Therefore, the aim of the present work was to develop a new polymeric composite post-reinforced with glass fiber, i.e., glass fiber-reinforced polybenzoxazine composites. The mechanical and thermal properties of the composites reinforced with various glass fiber contents were studied. The effect of composite glass fiber contents on the stress distribution of the tooth restored with the composite posts by finite element analysis using a commercial simulation program (ANSYS Workbench 2022 R1 software) was also evaluated and compared with the natural tooth.

## 2. Materials and Methods

### 2.1. Materials

Benzoxazine monomer (BA-a) was prepared from bisphenol A supported by PTT Phenol Co., Ltd., Rayong, Thailand, formaldehyde purchased from Merck Co., Ltd., Darmstadt, Germany, and aniline obtained from Panreac Quimica S.A., Barcelona, Spain. All chemicals were used as received. E-glass fiber plain fabrics with an areal density of 600 g/m^2^ were purchased from Thai Poly Add Ltd. Partnership, Bangkok, Thailand.

### 2.2. Benzoxazine Resin, Prepreg and Composite Manufactures

Benzoxazine monomer (BA-a) was prepared from bisphenol A, paraformaldehyde, and aniline based on the solventless method at a molar ratio of 1:4:2 [38]. A light yellow, low viscosity liquid monomer was produced by mixing the three reactants continuously for 40 min at 110 °C. The monomer then solidified at room temperature. The obtained solid monomer was ground into a fine powder and stored for later use in a refrigerator.

The molten BA-a monomer was pre-impregnated onto glass fiber plain fabrics at 80 °C. The composite laminates were preheated at 180 °C for 1 h and cured at 200 °C for 2 h using a pressure of 15 MPa in the compression molder, Labtech Engineering Co., Ltd., Samutprakarn, Thailand. After being allowed to cool to ambient temperature, the samples were ready for characterization.

### 2.3. Composition and Density Measurement

The experimental density of glass fiber-reinforced polybenzoxazine composites and unreinforced ones was measured according to ASTM D792–20 (Method A) by a water displacement method. The density of polybenzoxazine (poly(BA-a)) was also determined as a reference. The sample dimension was 25 mm × 50 mm × 3 mm. Each composite composition was tested for five samples and the average values were informed. The experimental density of the samples (*ρ*) is as follows:

(1) $$\rho =\frac{m_{a}}{m_{a}-m_{w}}\times \rho_{w}$$

When *m_a_* is the weight of the sample in air (g), *m_w_* is the weight of the immersed sample in water (g) and *ρ_w_* is the density of water (g/cm^3^).

### 2.4. Bending Test

The flexural properties of the samples were assessed using a universal testing machine, model 8872, Instron (Thailand) Co., Ltd., Bangkok, Thailand. The samples were examined in accordance with ASTM D790M-93 using a 48 mm support span and a 1.2 mm/min crosshead speed. The sample dimension was 25 mm × 50 mm × 3 mm. Each composite composition was tested for five samples and the average values were reported.

### 2.5. Dynamic Mechanical Analysis

Dynamic mechanical properties of the samples were obtained using a dynamic viscoelastic analyzer, model DMA1, Mettler-Toledo (Thailand) Ltd., Bangkok, Thailand. Three-point bending was employed throughout the test. The strain is measured at a frequency of 1 Hz with an amplitude of 30 μm. The sample was heated from 30 °C to 300 °C at a rate of 2 °C/min. The sample dimension was 10 mm × 50 mm × 3 mm. Three samples of each composite composition were replicated.

### 2.6. Scanning Electron Microscopy (SEM)

Scanning electron microscope (SEM), model JSM-6510A, JEOL Ltd., Tokyo, Japan was used to investigate the interfacial bonding of between glass fiber and polybenzoxazine matrix at a 20 kV acceleration voltage. To make the surfaces of all samples conductive, a thin layer of gold was applied using a sputtering apparatus, model SCD 040, Oerlikon Balzers Coating Co., Ltd., Chonburi, Thailand.

### 2.7. Finite Element Analysis (FEA)

**Figure 1:**
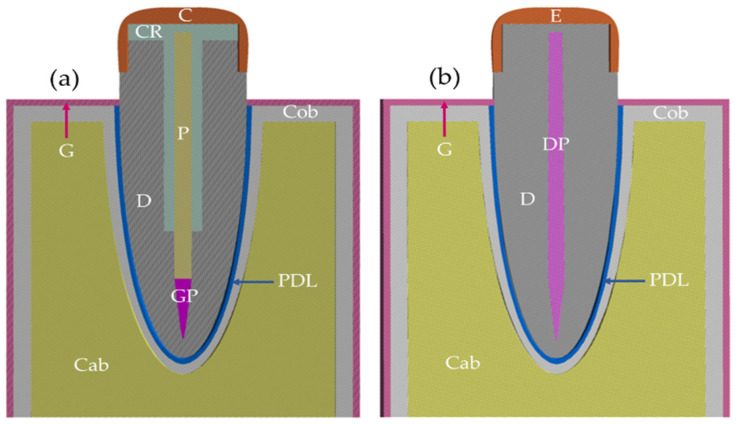
Schematic of tooth models: (**a**) tooth restored with glass fiber post (GF-reinforced poly(BA-a) composite, (**b**) natural tooth; C—Porcelain crown, CR—Composite resin, D—Dentin, DP—Dental pulp, P—Post, GP—Gutta-percha, E—Enamel, Cob—Cortical bone, Cab—Cancellous bone, PDL—Periodontal ligament.

Two 3D models were created in the Design Modeler of ANSYS Workbench 2022 R1 (File version 22.1.0.2021111419) software, Ansys Inc., Canonsburg, PA. The endodontically treated tooth model includes all structures (crown, composite resin, GF-reinforced poly(BA-a) composite post, dentin, dentin pulp, gutta-percha, enamel, cortical bone, cancellous bone, periodontal ligament) and natural tooth as can be seen in Figure 1.

The 3D FEM was analyzed by ANSYS Workbench 2022 R1 software. A 3D mesh was then crated with structurally solid elements defined by nodes. The number of elements and nodes generated by the tooth restored with post models was 112,376 and 208,652, respectively, while that of the natural tooth model was 110,535 and 204,398, respectively.

All structures and materials, i.e., crown, composite resin, dentin, dental pulp, gutta-percha, enamel, cortical bone, cancellous bone, and periodontal ligament were regarded as homogeneous, isotropic, and linearly elastic as listed in Table 1. Except for GF-reinforced poly(BA-a) composites acted as glass fiber posts are considered orthotropic material is known as a material having properties that differ along the three orthogonal planes as summarized in Table 2.

Based on previous studies [9,10,40], an occlusal load of 100 N was applied on the crowns axially at an angle of 45 degrees to the vertical axis for both models. The analyses of the stress distributions after simulating the models with the ANSYS software (ANSYS Workbench 2022 R1) of a tooth restored with glass fiber-reinforced poly(BA-a) composite posts were conducted according to von Mises criteria. For the quantitative analysis, the maximum von Mises stress values and patterns of teeth that had been restored with glass fiber-reinforced poly(BA-a) composite posts were assessed. The stress distributions were represented by color scheme maps, with the highest stress in red and the lowest stress in blue.

## 3. Results and Discussion

### 3.1. Density Measurement

**Figure 2:**
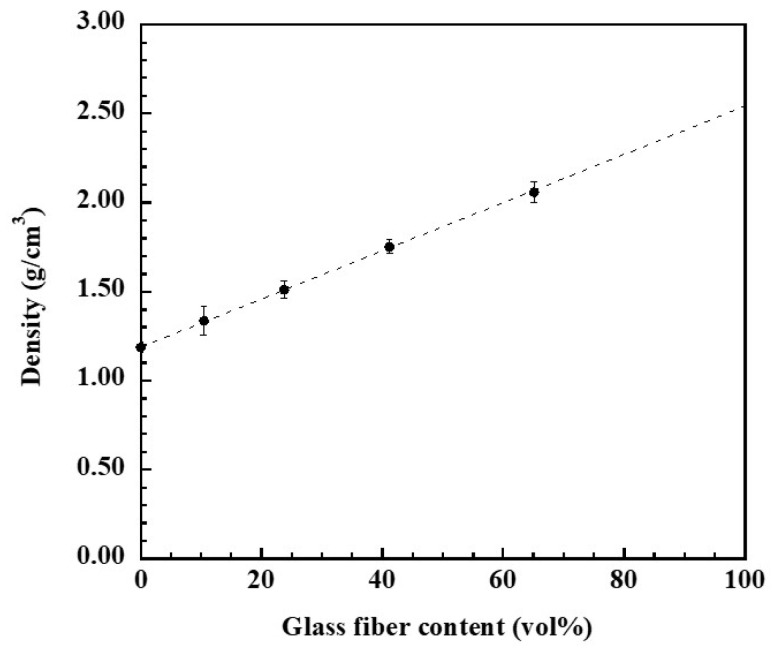
(⬤) Experimental density, (-----) theoretical density of GF-reinforced poly(BA-a) composites at various GF contents.

One of the most important aspects in defining the properties of composites is density, which is dependent on the relative quantity of reinforcing and matrix components. The density of glass fiber (GF)-reinforced polybenzoxazine (poly(BA-a) composites compared with unreinforced poly(BA-a) is plotted in Figure 2. It was found that the density of the GF-reinforced poly(BA-a) composites showed higher than that of the unreinforced poly(BA-a). This characteristic influenced from higher density of GF reinforcing, i.e., 2.54 g/cm^3^ [44] than the density of poly(BA-a), i.e., 1.19 g/cm^3^ [36]. In addition, to verify the density of the GF-reinforced poly(BA-a) composites, their experimental density was compared with their theoretical density calculated according to the rule of the mixture as follows Equation (2).

(2) $$\rho_{c}=\rho_{f}V_{f}+\rho_{m}V_{m}$$

When *ρ* and *V* is the density and the volume fraction, respectively. The subscripts *c*, *f* and *m* represent composite, fiber, and matrix, respectively.

The experimental density of the GF-reinforced poly(BA-a) composites tended to agree with their theoretical density until the poly(BA-a) composite was reinforced with 65.2 vol% GF (or 80 wt% GF), which was relatively high glass fiber content compared with other glass fiber-reinforced polymers, i.e., 36–60 vol% [26,27,28]. Due to an outstanding fiber wet-out using the low melt viscosity of the BA-a monomer, i.e., 1000 cP at processing temperatures [45,46], it is possible that voids were not formed in the poly(BA-a) composites reinforced with 10.5–41.3 vol% GF (or 20–60 wt%). While micro-void formation tended to form for the poly(BA-a) reinforced with 80 wt% as the slightly lower averaged experimental density than its theoretical density was observed. Therefore, it was expected that overall mechanical property such as flexural property of the poly(BA-a) composites reinforced with 10.5–41.3 vol% GF (or 20–60 wt% GF) showed no affect from void, while micro-void formation for the poly(BA-a) composite reinforced with 65.2 vol% GF (or 80 wt% GF) may result in insignificant decrease in flexural strength of the composite.

### 3.2. Mechanical Property

**Figure 3:**
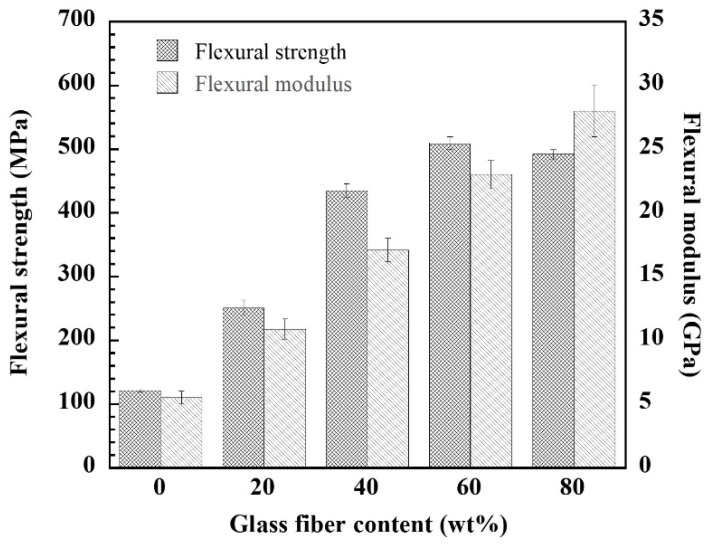
Flexural property of GF-reinforced poly(BA-a) composites at various GF contents.

Figure 3 shows mechanical properties, i.e., flexural modulus (elastic modulus under flexure mode) and flexural strength of the GF-reinforced poly(BA-a) composites compared with that of unreinforced poly(BA-a). The flexural modulus of the poly(BA-a) composites increased with an increase in GF content, i.e., 10.9, 17.1, 23.0, and 28.0 GPa for 20, 40, 60, and 80 wt% GF (or 10.5, 23.8, 41.3, and 65.2 vol% GF), respectively, and their flexural modulus was higher than that of unreinforced poly(BA-a), i.e., 5.50 GPa. The increment of the flexural modulus of the poly(BA-a) composites was due to the elastic modulus of the GF of about 70–85 GPa [47], which is higher stiffness than poly(BA-a) matrix. As is common knowledge, the stiffness of materials can be determined by their elastic modulus value, which is calculated as the ratio of stress to strain in an elastic condition. Therefore, to apply the GF-reinforced poly(BA-a) composite as a glass fiber post, it is possible that the 60 wt% GF- and 80 wt% GF-reinforced poly(BA-a) composites can be used. Since they showed an elastic modulus similar to that of dentin, i.e., 18– 40 GPa [9,10], which is a critical parameter for load transmission. This behavior is expected that can help potentially decrease the fracture risk of the remaining tooth structure. In addition, the flexural modulus of the GF-reinforced poly(BA-a) composites was in agreement with that of commercial glass fiber posts, i.e., a flexural modulus of 10–25.5 GPa [25].

Moreover, as expected, the flexural strength of the GF-reinforced poly(BA-a) composites showed higher than that of the unreinforced poly(BA-a). This is possible that the higher strength of the glass fiber, i.e., 3,300–3,450 MPa [44,48] than that of poly(BA-a), i.e., 121 MPa [49]. The flexural strength of the poly(BA-a) composites reinforced with 20–80 wt% GF was in a range of 250–504 MPa, which is the highest flexural strength owned to the poly(BA-a) composite reinforced with 60 wt% GF, i.e., 504 MPa. Theoretically, a variety of parameters, such as the fiber content, thickness, orientation, aspect ratio, bonding to the resin matrix, polymerization-induced stress, and variations in the intrinsic properties of the fibers and matrix, affect the mechanical properties of fiber posts [50]. Therefore, the enhancement of flexural strength of the 60 wt% GF-reinforced poly(BA-a) composite may indicate that good interfacial adhesion between poly(BA-a) and glass fiber was achieved. While, as also expected, the increase in the glass fiber content beyond 60 wt% resulted in a slight decrease in the flexural strength of the glass fiber-reinforced poly(BA-a) composite, i.e., 493 MPa. This may cause micro-void formation due to the reduction of the amount of polymer matrix wetting up the fibers. However, it is possible that the poly(BA-a) composites reinforced with 60–80 wt% GF can be acted as a glass fiber post since their flexural strength was higher than that of natural dentin at 212 MPa [51] and the flexural strength for root canal posts should be at least 400 MPa [20,26].

### 3.3. Dynamic Mechanical Analysis

**Figure 4:**
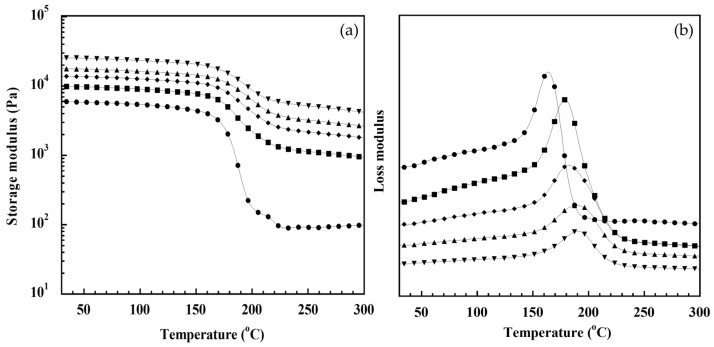
(**a**) Storage modulus (**b**) loss modulus of GF-reinforced poly(BA-a) composites at various GF contents: (⬤) 0 wt%, (■) 20 wt%, (◆) 40 wt%, (▲) 60 wt%, (▼) 80 wt%.

Dynamic mechanical properties, i.e., storage modulus and loss modulus as a function of the temperature of the GF-reinforced poly(BA-a) composites and unreinforced poly(BA-a) are depicted in Figure 4. The storage modulus that showed the material stiffness of the samples at a glassy state region reflecting their molecular rigidity, from the figure, revealed the storage modulus at 30 °C of the GF-reinforced poly(BA-a) composites were higher than that of the unreinforced poly(BA-a) as similarly observed in modulus under flexure mode. In addition, the storage modulus of each GF-reinforced poly(BA-a) composite was quietly stable before the storage modulus dropped sharply in the transition region, indicating that the reinforcement of poly(BA-a) by glass fiber can obtain the composites having more dimensional stability. The significant increase in rubbery plateau modulus of the GF-reinforced poly(BA-a) composites was also observed. The similar behavior was also reported in carbon fiber-reinforced polymer based on polybenzoxazine [52] and Kevlar-reinforced polybenzoxazine/polyurethane hybrid composites [53].

Moreover, the glass transition temperature (T_g_) of the GF-reinforced poly(BA-a) composites was determined using the peak positions of the loss moduli, and the T_g_ of the poly(BA-a) composites reinforced with 0, 20, 40, 60, and 80 wt% GF was 163, 178, 182, 190, and 192 °C, respectively, which was much higher than that of the conventional epoxy GFRP [54]. The substantial enhancement in the T_g_ of the poly(BA-a) composites reinforced with glass fiber is possible that glass fiber reinforcing hindered the movement of molecular chains of poly(BA-a). Good interfacial adhesion between glass fiber and poly(BA-a) matrix may also help promote the composite’s T_g_ improvement since high energy (high temperature) to move molecular chains is required.

### 3.4. Interfacial Adhesion between Glass Fiber and Polybenzoxazine

**Figure 5:**
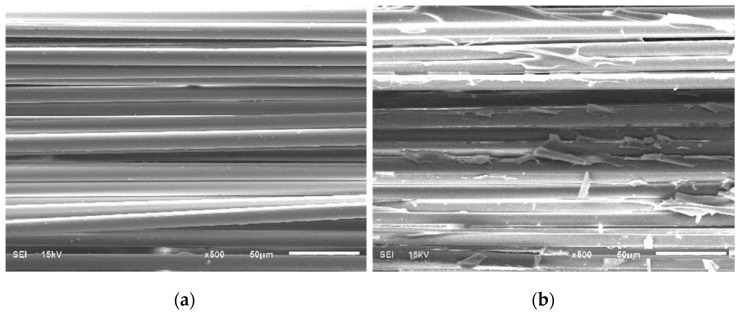
SEM micrographs (**a**) glass fiber morphology, (**b**) fractured surface of 80 wt% GF-reinforced poly(BA-a) composite.

To confirm that interfacial adhesion between poly(BA-a) and glass fiber (GF) is good, reflecting the good mechanical properties of the GF-reinforced poly(BA-a) composites was obtained. Scanning electron microscopic (SEM) technique is used to observe the interfacial adhesion between the glass fiber and the poly(BA-a) matrix. Figure 5 illustrates the surface morphology of glass fiber and the fractured surface of the GF-reinforced poly(BA-a) composite.

From Figure 5a, the relatively smooth, defect-free GF, and a diameter of about 10 μm of GFs’ cylindrical morphology was observed. The fractured surface of poly(BA-a) composite reinforced with 80 wt% is presented in Figure 5b. Due to the substantial interfacial adhesion between the glass fiber and matrix, the cohesive fracture surface developed in the poly(BA-a) matrix region which was observed as similarly discussed in carbon fiber-reinforced polybenzoxazine base [52] and GF-reinforced poly(BA-a) composite [55].

### 3.5. Stress Analysis Using Finite Element Analysis

**Figure 6:**
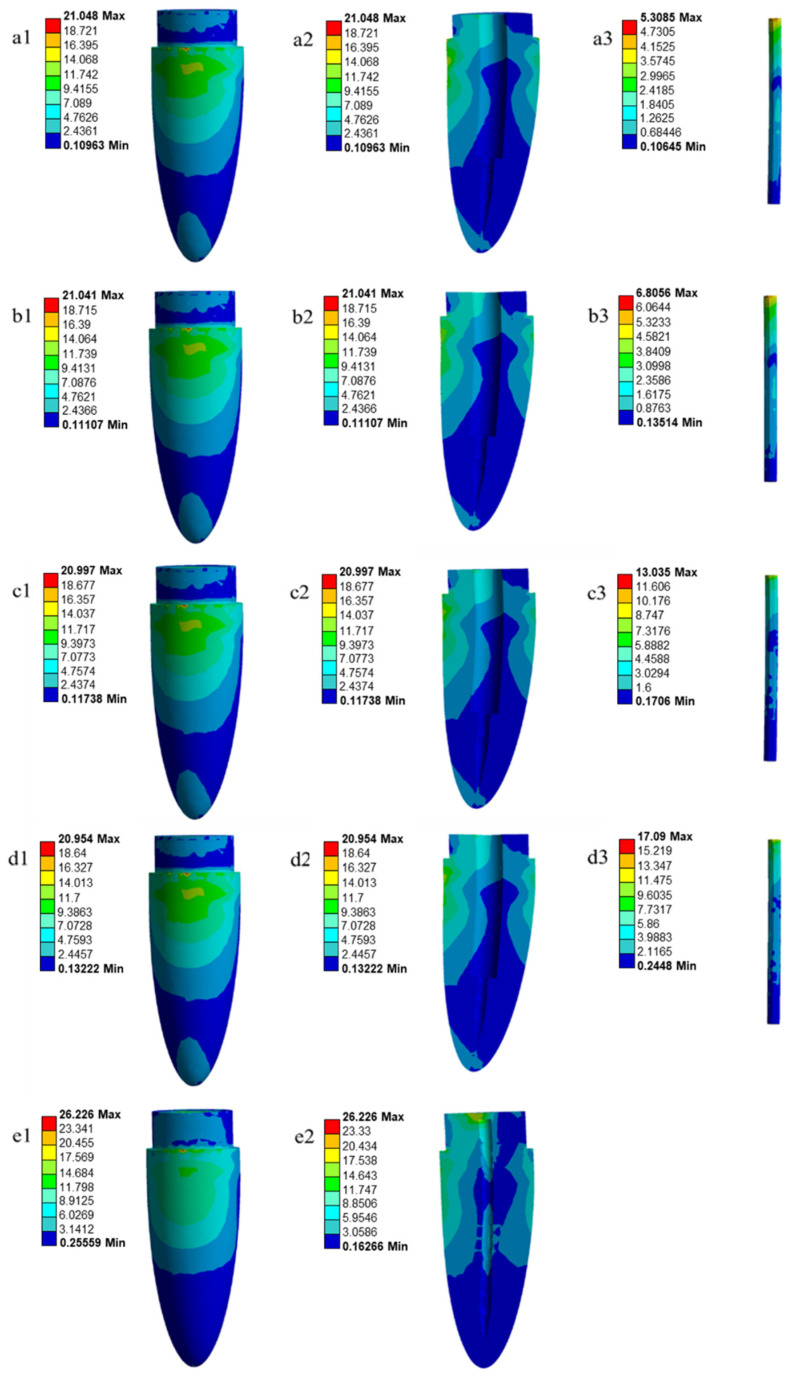
Contour map of the von Mises stress of tooth restored with GF-reinforced poly(BA-a) composites as a glass fiber post and natural tooth with 100 N applied load: GF-reinforced poly(BA-a) composite posts at various GF contents: (**a**) 20 wt%, (**b**) 40 wt%, (**c**) 60 wt%, (**d**) 80 wt%, (**e**) natural tooth.

The possibility to use GF-reinforced poly(BA-a) composites as glass fiber posts is estimated by finite element analysis since the post-performance response and process are complex, numerical simulations have been used to better understand the stress distribution and deformation that are created upon load applied. Von Mises stress by static analysis which gives the failure predilection of the material analyzed was investigated. Figure 6 presents the von Mises stress distribution in a tooth restored with GF-reinforced poly(BA-a) composite posts at various GF contents after an occlusion at 45 degrees oblique load of 100 N is applied. The load of 100 N was employed since it is the average load magnitude during normal chewing and a range of clinically relevant load application positions on the occlusal surface for the natural teeth of humans, i.e., incisor and first premolar [9,10,40]. From Figure 6a1–d1 and Figure 6a2–d2, the maximum equivalent (von Mises) stress occurred at the cervical region in dentin in all models of the tooth restored with GF-reinforced poly(BA-a) composite posts which were similarly seen in an endodontically treated tooth restored with fiber-reinforced composites, i.e., carbon fiber-reinforced PEEK and glass fiber-reinforced PEEK by a finite element analysis [21]. In addition, the stresses observed during oblique loading in the models were also agreed with the location of the fracture in fiber post and resin composite core-restored teeth subjected to fracture strength test [56] and with the in vitro mechanical test [57].

Furthermore, it was found that the elastic modulus of the GF-reinforced poly(BA-a) composite posts increased, i.e., 10.9, 17.1, 23.0, and 28.0 GPa, magnitude of the maximum equivalent (von Mises) stress in dentin decreased, i.e., 21.048, 21.041, 20.997, and 20.954 MPa for 20, 40, 60, and 80 wt% GF, respectively, as shown in Figure 6a1–d1. This characteristic correlated with the load fractions carried by the poly(BA-a) composite posts reinforced with 20, 40, 60, and 80 wt% GF, i.e., 5.3085, 6.8056, 13.035, and 17.090 MPa, respectively, as can be seen in Figure 6a3–d3. Therefore, from the results, the larger load fraction can be carried by the poly(BA-a) composite post reinforced with higher GF content which have an elastic modulus similar to that of dentin. The bending of the tooth restored with GF-reinforced poly(BA-a) composite post was also observed as can be seen in Figure 6a2–d2. It is possible that stress concentrations in the post cannot propagate directly into the dentinal region [40]. In addition, minimal color change at the apical third of the root means the complete dissipation of occlusal load, thereby decreasing the chance of vertical fractures.

To compare maximum equivalent (von Mises) stresses of the tooth restored with the GF-reinforced poly(BA-a) composite post with that of the natural tooth (Figure 6e1,e2), the maximum (von Mises) stresses of the endodontically restored tooth and the natural tooth were at the dentin region and cervical thirds of the root. In addition, teeth restored with 60 wt% and 80 wt% GF-reinforced poly(BA-a) composite posts still resemble the situation of a natural tooth due to minimized rigidity difference between the dentin and the composite posts. However, it is possible that 80 wt% GF-reinforced poly(BA-a) composite post is the material for the fabrication of endodontic post as it had generated less stress in the remaining dentin.

## 4. Conclusions

Highly filled glass fiber-reinforced polybenzoxazine composites for post-application were successfully developed. The effects of glass fiber contents on mechanical and thermal properties, and mechanical response to externally applied load by finite element analysis of glass fiber-reinforced polybenzoxazine composites can be summarized as follows. With outstanding property as low a-stage viscosity of benzoxazine monomer, the polybenzoxazine reinforced with relatively high glass fiber content when compared to almost commercial glass fiber polymer composites for posts was obtained. The flexural properties, i.e., flexural modulus and flexural strength of the glass fiber-reinforced polybenzoxazine composite showed quietly similar to that of dentin. This behavior indicated that the rigidity difference between the glass fiber-reinforced polybenzoxazine composite and the dentin which is a critical parameter for load transmission was minimized. The glass transition temperature of the glass fiber-reinforced polybenzoxazine composite was remarkably higher than that of the neat polybenzoxazine due to substantial interfacial adhesion between glass fiber and polybenzoxazine matrix. In addition, the tooth restored with the glass fiber-reinforced polybenzoxazine composite post provided a lower maximum stress value in the dentin than that in the dentin of the natural tooth.

## Figures and Tables

**Table 1:** Summarized elastic properties of the isotropic materials used for the FEA.

Material	Elastic Modulus (GPa)	Poisson’s Coefficient
Porcelain (crown) [28,39,40]	120	0.28
Composite resin [41,42]	16.6	0.24
Dentin [28,39]	18.6	0.31
Dental pulp [28,43]	0.002	0.45
Gutta-percha [28,39,40]	0.00069	0.45
Enamel [28,39]	41.0	0.30
Cortical bone [28,39]	13.7	0.30
Cancellous bone [39]	1.37	0.30
Periodontal ligament [39]	0.0689	0.45

**Table 2:** Summarized elastic properties for the orthotropic materials used for the FEA.

Elastic Constant	GF-Reinforced Poly(BA-a) Composite Posts at Various GF Contents			
	20 wt%	40 wt%	60 wt%	80 wt%
*E*_L_ (GPa)	10.9	17.1	23.0	28.0
*E*_T_ = *E*_T__′_ (GPa)	6.74	8.66	12.02	19.45
*G*_LT_ = *G*_LT__′_ (GPa)	2.37	3.01	4.22	7.28
*G*_TT__′_ (GPa)	2.53	3.27	4.63	7.26
ν_LT_ = ν_LT__′_	0.28	0.26	0.24	0.22
ν_TL_ = ν_T__′__L_	0.17	0.13	0.13	0.15
ν_TT__′_	0.33	0.33	0.30	0.25

*E*_L_ obtained from experimental data as reported in item 3.2, while the other values are theoretical values calculated from relationships as shown in Appendix A in Ref. [28].

## Data Availability

Data is contained within the article.
